# Cushing’s disease: a multidisciplinary overview of the clinical features, diagnosis, and treatment 

**Published:** 2016

**Authors:** A Buliman, LG Tataranu, DL Paun, A Mirica, C Dumitrache

**Affiliations:** * Titu Maiorescu University, Faculty of Medicine, Bucharest, Romania; ** ”Carol Davila” University of Medicine and Pharmacy, Bucharest, Romania; *** Bagdasar-Arseni Clinical Emergency Hospital, Bucharest, Romania; **** C.I. Parhon National Institute of Endocrinology, Bucharest, Romania

**Keywords:** pituitary adenoma, adrenocorticotropic hormone, corticotrophin-releasing hormone, ACTH hypersecretion, Cushing’s disease

## Abstract

Cushing’s disease is considered a rare condition characterized by the hypersecretion of the adrenocorticotropic hormone (ACTH) due to a pituitary adenoma that ultimately causes endogenous hypercortisolism by stimulating the adrenal glands. The clinical signs suggesting Cushing’s disease, such as obesity, moon face, hirsutism, and facial plethora are already present on presentation. Endogenous hypercortisolism is associated with an increased risk of cardiovascular and metabolic manifestations, as well as respiratory disorders, psychiatric complications, osteoporosis and infections, leading to high rates of morbidity and mortality. It is vital to diagnose Cushing’s disease as early as possible and to implement a treatment plan to lead to a successful prognosis and a low number of complications.

The goal of this article was to review the clinical, diagnostic and treatment aspects of Cushing’s disease using the most recent available guidelines.

## Case report

Cushing’s disease is caused by endogenous hypercortisolism. This is due to the hypersecretion of the adrenocorticotropic hormone (ACTH) by an ACTH-secreting pituitary adenoma [**1**].

It is considered a rare condition, though it is recognized as a cause of Cushing’s syndrome [**2**].

The ACTH-secreting pituitary adenoma, along with the unsuppressed cortisol hypersecretion, also produces bilateral adrenocortical hyperplasia [**3**]. 

In most cases, the anatomopathological examination shows a basophilic or a chromophobe pituitary adenoma (especially the larger ones) [**1**]. 

The prevalence of Cushing’s disease is of 40:1,000,000 people and more often occurs in women (sex ratio of 9:1 in favor of women) [**1**]. Of all pituitary adenomas, functional and non-functional, the ACTH-secreting adenoma represents about 10–12% [**1**], 5.3% [**4**,**5**,**6**].

Cushing’s disease is associated with an increased risk of cardiovascular and metabolic manifestations, as well as respiratory disorders, psychiatric complications, osteoporosis and infections leading to high rates of morbidity and mortality [**2**]. Because these are a consequence of endogenous hypercortisolism, which is associated with a high rate of morbidity even after a favorable treatment, it is vital to diagnose Cushing’s disease as early as possible and to implement a treatment plan to lead to a successful prognosis and a low number of complications. 

## Clinical presentation

On presentation, over 50% of the patients with Cushing’s disease have pituitary microadenoma with a diameter smaller than 5 mm, which are difficult to see through imaging investigation (Computer Tomography and Magnetic Resonance) [**1**]. Of these, only 10% are large enough to produce a mass effect on the cerebral tissue or to affect the structure of the sellar region (ballooned or increased in size or contour of the sella turcica) [**1**].

Clinical signs and symptoms of an ACTH-secreting pituitary adenoma are caused by the tumor compression (which rarely occurs, due to the tumor’s small size) and by cortisol and androgen excess (Cushing’s disease). The latter include:

• • weight gain, generalized in 50% of the cases [**1**] or sometimes with centripetal fat distribution, especially on the trunk, abdomen, interscapular (“buffalo hump”) supraclavicular fat pat, round plethoric face (“moon face”) [**1**,**2**,**3**] 

• • hypertension 

• • thin skin, easy bruising, capillary fragility, purplish-red striae (thighs, flanks, lower abdomen, upper limb root, breasts), acne, flushing, fungal skin infections, poor healing of skin wounds [**7**]

• • lower limb edema 

• • hypotrophy and proximal limb muscle fatigue [**2**] 

• • impaired glucose tolerance or type 2 diabetes [**8**] 

• • osteopenia or osteoporosis with pathological vertebral compression fractures, aseptic necrosis of the femoral head 

• • hyperpigmentation of the skin and mucous membranes, a consequence of the ACTH-MSH cross reactivity, which occurs in high levels of ACTH (in Cushing’s disease and non Cushing’s syndrome) or in the secretion of ectopic ACTH (also Nelson syndrome) [**1**] 

• • manic-depressive psychosis, depression, emotional debility, dementia [**9**] 

• • secondary amenorrhea, hirsutism, decreased sexual dynamic 

• • leukocytosis, lymphopenia, eosinopenia, decreased immunity and frequent infections 

These clinical manifestations may vary from patient to patient. Some are common to other diseases, such as obesity, Cushing’s syndrome, pseudo-Cushing’s syndrome or other ectopic ACTH secretion.

Cushing’s disease is associated with increased rates of morbidity and mortality, due to systemic glucocorticoid excess [**3**].

## Paraclinical investigations

### Screening tests

Screening tests are used in Cushing’s disease to identify the excessive secretion of cortisol, loss of diurnal variation of ACTH and cortisol and damaged negative feedback mechanism, involved in the pituitary-adrenal axis. The confirmation tests or imaging investigations are needed to locate the lesion in the diagnosis of Cushing’s disease. It is also important to perform the confirmation and screening tests in stress-free conditions, avoiding non-specific stimulation of the pituitary-adrenal axis. Secondary to steroid hormone administration, Iatrogenic Cushing’s syndrome should be taken into consideration and excluded.

#### 1. Testing diurnal variation in ACTH and cortisol levels

Cortisol is secreted by the adrenal gland in a pulsatile manner accompanying the pulsatile secretion of ACTH. The peak cortisol secretion was registered at 8:00 AM and had a value of 6.5 to 25.4 µg/ dL with a range of 180–700 nmol/ L. The minimum peak was recorded at 00:00 with a value of less than 3,6 mg/ dL (100 nmol/ L). Random blood sampling was not recommended, as diurnal variation in blood of normal individuals was absent in patients with Cushing’s disease. The repetition of the tests for several times was also recommended [**10**].

#### 2. Testing urinary free cortisol over 24 hours (UFC)

The sensitivity and specificity of this test in hospitalized patients was higher than the “Overnight” Dexamethasone 1 mg test. Dosage involved methods of radioimmunoassay, HPLC (High Performance Liquid Chromatography) or mass spectrometry. The normal values of urinary free cortisol levels obtained by using radioimmunoassay were above 90 mg/ 24h and 50 mg/ 24h by using HPLC. Values exceeding two to three times the upper normal limit were indicative of Cushing’s syndrome. The method cannot be used in diagnosing adrenocortical insufficiency, because low cortisol excretion/ levels in the urine can also be found in healthy people. It is, therefore, advisable to measure urinary creatinine and volume to evaluate its quality [**11**,**12**,**13**].

#### 3. Testing serum and salivary cortisol by night

Under physiological conditions, serum cortisol concentration reached a maximum peak by midnight. There was a strong correlation between the values of serum and salivary cortisol, which facilitated their determination in stress-free conditions.

Harvesting midnight serum cortisol was done while the patient was asleep or awake by placing an intravenous catheter before bedtime and after at least 48 hours’ hospitalization. A serum cortisol value higher than 5 mg/ dL (140nmol/ l) while the patient was asleep or higher than 7.5 mg/ dL (207nmol/ l) (3.13) when awake, characterized Cushing’s syndrome. When a value was lower than 1.8 mg/ dl (50nmol/ l) it ruled out the diagnosis.

Salivary cortisol determination was executed through radioimmunoassay and expressed free fraction of cortisol. A value higher than 0.13 mg/ dL [**14**,**15**] (at two different determinations) of salivary cortisol between 23:00 and 0:00 PM proved to be a test with a sensitivity and specificity of 95% regarding the identification of patients with Cushing’s syndrome.

#### 4. “Overnight” Dexamethasone 1mg

The “overnight” test was used as a screening test in highlighting the unsuppressed hypercortisolism regardless the cause [**15**,**16**]. One mg Dexamethasone was administered between 23:00–24:00 PM followed by the determination of serum cortisol next day at 08:00 AM. Values exceeding 1.8 mg/ dL (50 nmol/ L) represented a positive diagnosis of Cushing’s syndrome. Cases presenting as obese patients or as those suffering from depression should be taken into consideration, because cortisol was not suppressed.

## Diagnostic tests

### 1. Evaluation of the sellar region by using magnetic resonance investigation

This investigation was critical in the diagnosis of Cushing’s disease because it evaluated the preoperative anatomic structure of the pituitary gland and confirmed the presence or absence of a pituitary adenoma. There is a strong correlation between the MRI aspects of ACTH-secreting pituitary adenomas (86%) [**17**] and the intraoperative findings (77.8%) [**18**]. Most cases of Cushing’s disease were caused by pituitary macroadenomas that could be visualized by using radiological imaging [**18**,**19**]. MRI with contrast administration (0.05 mmol/ kg Gadolinium) was recommended because pituitary adenomas have a homogeneous enhancement, which distinguishes them from the normal pituitary tissue [**20**,**21**].

### 2. IPSS with Desmopressin or CRH

This test involved injecting, at the same time, the inferior petrous sinuses bilaterally and the peripheral blood, with Desmopressin or CRH and subsequently dosing basal ACTH [**22**]. An ACTH petrous sinus ratio/ peripheral ACTH > 2 before the administration and a petrous sinus ratio/ peripheral ACTH > 3 after the administration indicated Cushing’s disease [**23**,**24**]. A gradient below 1.4 indicated an ectopic ACTH source [**23**,**24**].

### 3. Overnight Dexamethasone 8 mg

This test was used to distinguish Cushing’s syndrome from the ectopic ACTH secretion. It involved the administration of 2 mg of Dexamethasone at every 6 hours, or a single dose of 8 mg at 23:00 PM, followed by the determination of serum cortisol the next day at 08:00 AM [**25**]. An increased secretion of cortisol indicated an ectopic source of ACTH, whereas a low secretion would confirm the diagnosis of Cushing’s disease [**26**,**27**].

## Treatment

Cushing’s disease treatment aims to improve clinical manifestations by the tumor mass resection with the decompression of the optic nerve and chiasm, normalizing the hormone secretion in both the hormonal hypersecretion and insufficiency, obtaining an anatomopathological confirmation and, not least, reducing the rate of recurrence. At the same time, the preservation of the normal surrounding tissue integrity should be followed, while avoiding the possible complications.

The current treatment and management of the pituitary adenomas is multimodal, including elective surgery, radiation, and drug therapy as supporting treatments. As a last step, bilateral adrenalectomy could be considered in treating the Cushing’s disease.

### Surgical therapy

Over 90% of the pituitary adenomas can be resected by using transsphenoidal surgery, associated with a complete remission rate of 60–90% for microadenomas and < 65% for macroadenomas [**28**,**30**,**33**]. Although this microsurgical approach is the most frequently used in treating Cushing’s disease, the endoscopic techniques have proven to have a significant impact in recent years and are increasingly used [**29**].

Comparing postoperative outcomes between the two surgical methods, no major differences have been reported [**30**]. The elective treatment for microadenomas, which are not large enough to be visible on imaging investigations, is the resection via total or partial hypophysectomy, with a relatively low (70%) remission rate, but with an increased frequency of hypopituitarism and postoperative complications [**31**,**32**].

The quality of tumor resection is expressed as it follows: total ablation (100%), near total ablation (> 90%), subtotal ablation (80–90%), partial ablation (< 80%) or biopsy [**4**,**5**]. The surgical resection depends on several factors: tumor size, invasion of the cavernous sinus, preoperative medical treatment (somatostatin and bromocriptine which modify tumor consistency), and the surgeon’s experience.

Biochemical remission is considered when serum cortisol is < 5 mg/ dL and urine is normalized. Most patients with Cushing’s disease require hormone replacement with an optimal dose of 12–15 mg/ m2 of glucocorticoid to correct hypercortisolism. The hormone replacement can be stopped when the serum cortisol level is > 18 mg/ dL. 

The recurrence rate for microadenomas, resected by transsphenoidal approach is of 5–10%, 5 years after surgery and of 10–20%, 10 years postoperatively. A higher recurrence rate was recorded among younger patients (< 25 years). Macroadenomas have a recurrence rate of 12–45%, and a higher and faster rate of occurrence (49 months versus 16 months), compared with microadenomas.

### Medical treatment

Medical treatment is a supporting treatment (similar with radiation therapy), while surgery remains the preferred treatment.

Drug treatment is recommended before pituitary surgery, in addition to radiotherapy or in cases in which surgery and radiotherapy are contraindicated.

Several classes of therapeutic agents are described to inhibit the adrenal steroidogenesis. They are indicated for the relief of preoperative effects such as hypercortisolism or unresectable pituitary tumors. These are Metoripon, Ketoconazole, Aminoglutethimide, Mitotane and Etomidate. Of these, the most commonly used in practice are the following:

• • Ketoconazole, in doses between 400–1200 mg/ day, progressively increasing dosage and monitoring the adverse effects (gastrointestinal disturbance, gonadal dysfunction and hepatic cytolysis) [**34**] 

• • Metopiron in doses between 1–4 g/ day, which can present side effects such as hypertension, ataxia, dizziness, lethargy and rash [**35**] 

• • op’DDD or Mitotane, which is an adrenolytic agent used in doses between 6–12 g/ day. A dosage near the limit of toxicity is used for effectiveness. At the same time, a close supervision of Mitotane blood levels and the prevention of acute addisonian decompensation are required. Adverse reactions consist of hypothyroidism, hypogonadism, digestive disorders, and ataxia [**36**]. 

The therapeutic agents with a direct effect on the pituitary gland are another class of drugs. Some drugs have proven to be ineffective in the treatment of corticotroph adenoma following numerous clinical and pharmacological trials, including Bromocriptine, Cyproheptadine and Valproate. 

Somatostatin analogues (Octreotide, Pasireotide) and dopamine agonists (Cabergoline) proved to be useful in the treatment of Cushing’s disease. Cabergoline therapy in doses between 1–7 mg per week, revealed a significant clinical improvement of symptoms and a decreasing level of urinary free cortisol in 24 hours [**37**]. 

Pasireotide has a high affinity for somatostatin receptor type 5 (SSTR 5), blocking the release of adrenocorticotropic hormone (ACTH). It turned out that Pasireotide is 40 times more efficient than Octreotide [**38**,**39**]]. The initial recommended dose of Pasireotide is of 0.6 mg, administered subcutaneously, twice daily, while monitoring the potential side effects, such as hyperglycemia, cholelithiasis, and digestive disorders [**40**].

Mifepristone is an anti-progestin agent (which is used to induce abortion). It may also have therapeutic uses in the treatment of endometriosis and uterine leiomyomas. In much higher doses, it acts as a glucocorticoid antagonist. In some studies, Mifepristone has shown to be effective in the treatment of some Cushing’s disease symptoms, especially in cortisol-induced psychosis [**41**]. In the US, a dose of 300 mg Mifepristone daily is indicated in the management of hyperglycemia, secondary to hypercortisolism, in adult endogenous Cushing’s syndrome, in which surgery is contraindicated.

Anticoagulation therapy is also recommended preoperatively, since hypercoagulability is known to characterize this pathology.

### Radiation

Radiotherapy after surgery is recommended in patients who present a tumor residue, especially if it tends to increase, or in cases in which pathological hormonal hypersecretion persists. 

Radiotherapeutic methods include fractionated radiotherapy, stereotactic radiosurgery (GammaKnife), CyberKnife, and Proton Beam Therapy.

#### 1. Fractionated External Beam Radiotherapy

Fractionated radiotherapy effects can be seen after an interval of two years and improvements are only visible in 50–70% of the patients after a period of 3–5 years [**42**]. Hypopituitarism can be seen in 40% of the patients and is followed by secondary brain tumors in 1–2% of the patients [**42**].

#### 2. GammaKnife

GammaKnife radiosurgery effects can be observed at 6 months after radiation. Remission is registered in 70–95% of the patients with Cushing’s disease [**43**]. Hypopituitarism occurs in 30% of the patients [**43**].

### Management of residual or recurrent Cushing’s disease

Surgical therapy remains the gold standard in treating residual or recurrent adenoma; radiotherapy, medical treatment, and bilateral adrenalectomy are alternative therapies. In symptomatic cases of Cushing’s disease, the generally favored approach is to perform transsphenoidal surgery as quickly as possible. An alternative approach is to delay surgery for 4-6 weeks, as some patients experience a progressively favorable outcome after surgery. In these cases, transsphenoidal surgery has a success rate of 50–70% and can increase in microadenomas.

### Prognosis and complications

Cardiovascular complications, low bone density, hypercoagulability, psychiatric and cognitive disorders are only partially alleviated in patients with Cushing’s disease under treatment. The cardiovascular risk is higher compared to normal people, even 5 years after a complete remission. The quality of life continues to deteriorate even after remission and tends to be lower in patients with hypopituitarism.

### Comorbidities and mortality

The mortality rate in patients with Cushing’s disease is 4 times higher than the general population [**44**,**45**]. The exposure to high levels of cortisol is associated with an increased rate of mortality due to metabolic, cardiovascular, psychiatric disorders or infections [**46**].

Cushing’s disease is also characterized by the frequent occurrence of osteoporosis (40%) and fractures, regardless of age, frequently associated with vertebral compression and spinal injuries. It is, therefore, important to incorporate a long-term action plan for the treatment of osteoporosis in patients with Cushing’s disease [**47**].

Hypercortisolism causes insulin resistance and impaired glucose tolerance, leading to diabetes mellitus in patients with Cushing’s disease (20–50%) [**48**].

Dyslipidemia also occurs, along with diabetes mellitus, both of which represent cardiovascular risk factors. Cardiovascular diseases are a common cause of death among patients with Cushing’s disease.

Complications associated with severe infections are reported in 42% of the patients [**48**].

## Conclusions

Cushing’s disease is caused by a pituitary ACTH-secreting adenoma, which stimulates the adrenal glands, causing endogenous hypercortisolism. This endogenous effect is accompanied by a series of cardiovascular complications, metabolic, psychiatric disorders, diabetes mellitus, and low bone density, all resulting in increased morbidity and mortality rates.

**Fig. 1:**
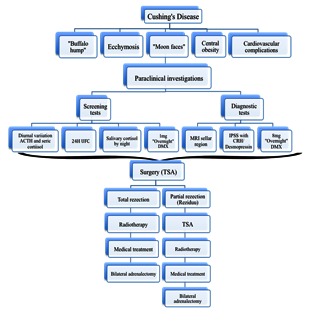
**Fig. 1**The multimodal management of Cushing’s disease according to the new guidelines

A brief summary of the multimodal management according to the new guidelines is presented in **Fig. 1**.

### Acknowledgement

This paper was cofinaced from the European Social Fund through Sectoral Operational Programme - Human Resources Development 2007-2013”, project number POSDRU/1871.5/S/155605, entitled “Scientific excelence, knowledge and innovation through doctoral programs in priority areas”, Beneficiary – University of Petrosani.
